# Iron Pill-Induced Gastritis in the Paediatric Population

**DOI:** 10.1155/2019/7527608

**Published:** 2019-09-11

**Authors:** Daniel Ching, Cathy Mews, Charles Crompton, Madhur Ravikumar, Disna Abeysuriya

**Affiliations:** ^1^Department of Anatomical Pathology, PathWest Laboratory Medicine, Perth Children's Hospital, Nedlands, Australia; ^2^Department of Gastroenterology, Perth Children's Hospital, Nedlands, Australia; ^3^Department of Nephrology, Perth Children's Hospital, Nedlands, Australia

## Abstract

Iron is the most common trace mineral in the body. The effects of iatrogenic iron pill-induced gastritis (IPIG) at therapeutic levels are underreported and underappreciated in the paediatric population. Herein, we report a case of an 11-year-old boy presenting with increasing epigastric pain and refusing oral intake secondary to iron pill tablets. We report only the second confirmed case of a paediatric patient with IPIG in the peer-reviewed literature.

## 1. Introduction

Iron is the most common trace mineral in the body and is a necessary compound for haemoglobin and myoglobin production. The absorption of iron, although not completely understood, is thought to occur in the duodenum and jejunum. The World Health Organization global prevalence report on anaemia in 2011 estimated that it affects 43% or 273 million children worldwide [1]. Iron deficiency anaemia (IDA) can lead to multisystemic disorders, notably irreversible neurocognitive developmental effects [2].

Oral iron supplementation in the form of ferrous sulfate is the most common treatment and like most medications is not free of side effects. At therapeutic levels, it is well known to cause gastropathy. The ferrous iron and ferric iron are catalysts for formation of reactive oxygen metabolites leading to local damage [3]. The effects of unintentional acute iron toxicity in the paediatric population are relatively common due to the widespread availability and their candy-like appearance of the tablets. However, the effects of iatrogenic iron pill-induced gastritis (IPIG) at therapeutic levels are underreported and underappreciated in the paediatric population. Herein, we report a case and review the literature.

## 2. Case Presentation

An 11-year-old boy presented to the emergency department with ongoing increasing epigastric pain and refusing oral intake of 2 week duration. The patient had received a renal transplant 3 years ago for end-stage renal failure secondary to posterior urethral valves and has since been on immunosuppressive therapy and remained under close surveillance. He was commenced on oral iron tablets (ferrous sulfate 325 mg/sodium ascorbate 562.4 mg modified release, once daily) for treatment of IDA about two months prior to this presentation. The blood results a week prior to endoscopy showed Hb 104 (125–170 g/L), haematocrit 0.34 (0.37–0.49 L/L), mean cell volume 100 (78–94 fL), ferritin 158 (20–100 *μ*g/L), iron 5 (7–25 *μ*mol/L), transferrin 31 (20–41 *μ*mol/L), and transferrin saturation 8% (12–45%).

**Figure 1:**
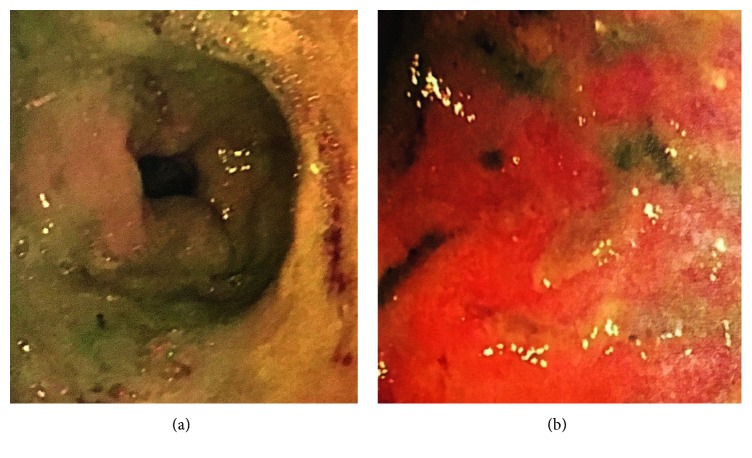
Endoscopy showed patchy redness and thickened gastric folds including an exudate involving the gastric antrum and the first part of the duodenum.

**Figure 2:**
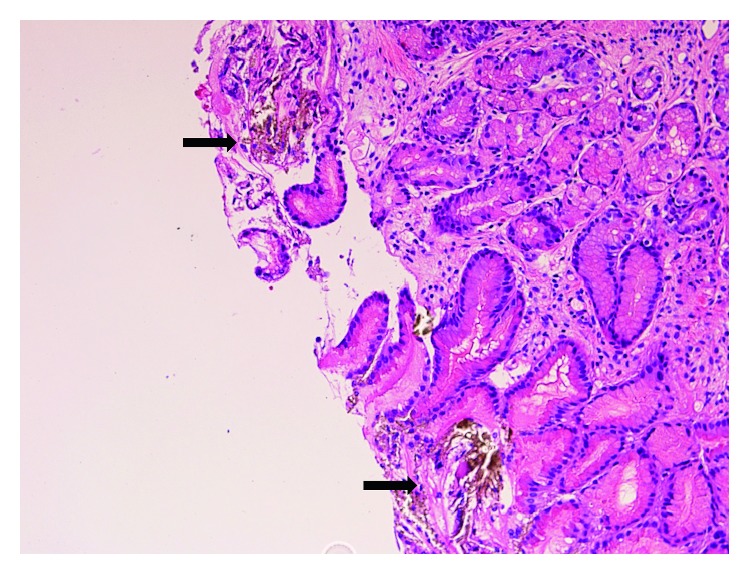
H&E showed focal areas of sloughed epithelium, refractive brown deposits (black arrows), and collections of inflammatory cells within the disrupted gastric pits.

**Figure 3:**
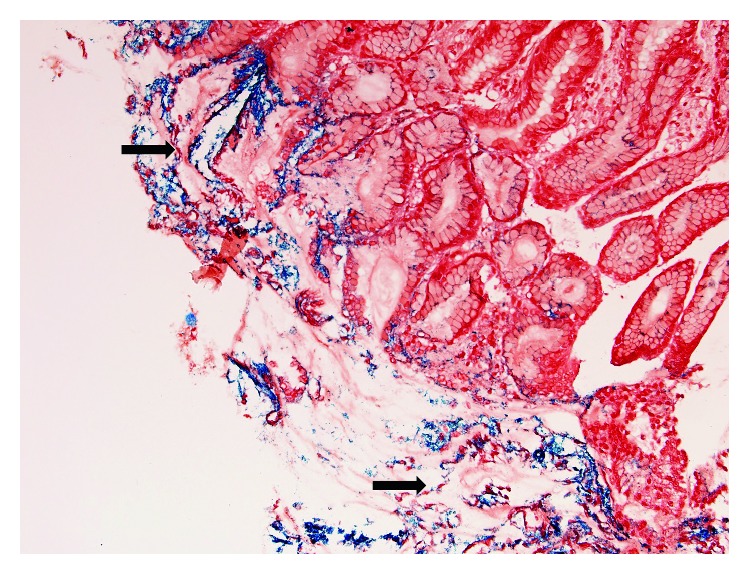
Special staining with PBR (black arrows) confirmed the refractile brown material to be iron deposits.

Endoscopy showed patchy redness and thickened gastric folds including exudate involving the gastric antrum and the first part of the duodenum (Figure 1). Serial biopsies were taken. Histopathological examination showed focal areas of sloughed epithelium associated with mucinous material and focal collections of lymphocytes and eosinophils within some disrupted gastric pits (Figure 2). Refractile brown deposits were seen on the surface mucosa and within the gastric pits. Prussian blue (PBR) staining confirmed it to be iron deposits (Figure 3). No *Helicobacter pylori* organisms were identified, and the remaining biopsies were unremarkable. The patient was diagnosed with IPIG. Iron supplements were immediately ceased, and he was commenced on omeprazole 20 mg twice daily. The patient was also given an iron infusion prior to discharge with a view of further infusions as needed. The symptoms resolved within 3 days, and a follow-up 4 weeks later confirmed complete resolution.

## 3. Discussion

Iron accumulation in the gastric mucosa is known as gastric siderosis (GS) and was first described in the literature in the 80s [4]. GS has been shown to be associated with oral iron supplements, blood transfusions, haemochromatosis, alcohol abuse, and hepatic cirrhosis. The stomach is not involved in iron metabolism; hence, the presence of GS without possible explanations should raise the need for further investigation and may be the initial presentation for underlying occult conditions.

The most common cause of GS is due to oral iron tablets which are well known to cause gastropathy and is termed IPIG. One study has shown that, of the 59 cases identified with iron deposition, 58 were on iron tablets of which 31 showed evidence of gastropathy [5].

However, the exact pathogenesis is poorly understood. Therapeutic levels of iron are thought to cause gastropathy via two theories: (1) due to the impact of tablets causing local effects mimicking a chemical burn, a process which can be generalised to other corrosive oral medications; and (2) could be associated with passive concentration-dependent uptake associated with long-term iron intake and overload [6]. Such effects could be further exacerbated by underlying gastrointestinal disorders and importantly the two processes may not be mutually exclusive.

The prevalence of oral IPIG is reported to be 0.7% in the adult population [7], with studies showing that up to 16% of patients on iron tablets is found to have detectable iron on tissue biopsy [5].

The diagnosis of IPIG requires endoscopy and histopathological examination. The clinical manifestations are often nonspecific with features of abdominal pain, nausea, and vomiting. Suspicion in the younger paediatric population is considered even more challenging due to the difficulty in obtaining an accurate clinical history.

Endoscopic findings have been described as yellow/brown mucosal discolouration, erythema, ulceration, and regenerative polyps [8, 9]. From the histopathological perspective, it is worth describing the exact site of involvement. GS can be seen in the mucosa or lamina propria, can be either intracellular or extracellular, and may even involve glandular structures. These pigments are often granular/fibrillary and refractile but nonpolarizable [7]. It is often accompanied by surrounding inflammation and reactive/regenerative changes. However, from our experience, these findings can be subtle and often require detail examination including serial sections.

The location of GS has become topical in recent years after Marginean et al. [10] classified it into 3 separate patterns: (A) iron deposition in macrophages, stroma, and epithelium; (B) predominantly extracellular iron deposition with focal involvement of blood vessels, macrophages, and epithelium; (C) iron deposition mostly in glandular epithelium of antral and fundic cells. Pattern A was described as nonspecific and is closely associated with inflammation and ulceration. All cases with pattern B were associated with oral iron supplements and known as iron-pill GS. Pattern C was relatively independent and closely associated with systemic conditions secondary to iron overload. Some authors argue that patterns A and B have somewhat similar mechanisms and overlapping histopathological features but for practical reasons will not be discussed here [11].

Of the different iron tablet preparations, ferrous sulfate is thought to be associated with most gastrointestinal side effects [7]. In minor cases, it would be worth considering different oral preparations including liquid forms and altering dosage and/or frequency. Some adult studies have suggested that IPIG is exclusive to tablet preparation that can be reversed when changing to liquid forms [12]. Such findings may be true in the adult population but has not been proven among the paediatric group.

Administration of gastric acid reduction agents such as protein pump inhibitors (PPI) or histamine H2-receptor antagonists is helpful. Kaye et al. [5] found an association between PPI use and GS in the adult population, but it is unclear if the PPI use was due to gastropathy or alkaline environment secondary to PPI use increases iron deposition. The sparsity of literature precludes evaluation of the treatment implications in the paediatric population.

It is also worth noting that gastropathy secondary to iron supplementation is not exclusive to the stomach and can occur in the duodenum as demonstrated in our patient. Mahjoub et al. reported the first case series of duodenal iron deposition in infants [13]. The use of iron supplementation should not be viewed lightly. It should be monitored regularly for therapeutic response and side effects. In cases with features of IPIG, further investigation may be warranted. Similar to many other paediatric conditions, safety-net advice to the patient and/or parents may be the simplest yet the most effective option for early identification of IPIG symptoms.

Almost all the published literature on therapeutic iron pill-induced gastritis is in adult population, and whether these findings can be extrapolated to the paediatric group is unknown. To the best of our knowledge, the only paediatric report was that of a 14-year-old girl reported by Meliţ et al. [14]. We report only the second confirmed case of a paediatric patient with IPIG in the peer-reviewed literature. IPIG should not be taken lightly and if not identified early, can lead to malnutrition, failure to thrive, and developmental delay with severe forms leading to haemorrhage. Clinicians should be aware of this rare complication of oral iron supplementation. Taking into consideration the large paediatric population on iron supplements, it is postulated that this occurrence may be more common than reported.

In conclusion, IPIG is not exclusive to adults. The use of iron supplementation can have significant consequences and should be regularly monitored in the paediatric population.
